# Prognostic Value of Axillary Lymph Node Texture Parameters Measured by Pretreatment ^18^F-Fluorodeoxyglucose Positron Emission Tomography/Computed Tomography in Locally Advanced Breast Cancer with Neoadjuvant Chemotherapy

**DOI:** 10.3390/diagnostics12102285

**Published:** 2022-09-22

**Authors:** Jae Pil Hwang, Joon Young Choi, Joon Ho Choi, Young Seok Cho, Sung Mo Hur, Zisun Kim, Cheol Wan Lim, Seongho Seo, Ji Eun Moon, Sang-Keun Woo, Jung Mi Park

**Affiliations:** 1 Department of Nuclear Medicine, Soonchunhyang University Bucheon Hospital, Bucheon 14584, Korea; 2 Department of Nuclear Medicine, Samsung Medical Center, Sungkyunkwan University School of Medicine, Seoul 06351, Korea; 3 Department of Surgery, Soonchunhyang University Bucheon Hospital, Bucheon 14584, Korea; 4 Department of Electronic Engineering, Pai Chai University, Daejeon 35345, Korea; 5 Department of Biostatistics, Soonchunhyang University Bucheon Hospital, Bucheon 14584, Korea; 6 Division of Applied RI, Korea Institutes of Radiological and Medical Sciences, Seoul 01812, Korea

**Keywords:** locally advanced breast cancer, neoadjuvant chemotherapy, positron emission tomography, texture analysis

## Abstract

Background: This study investigated the prognostic value of axillary lymph node (ALN) heterogeneity texture features through ^18^F-fluorodeoxyglucose positron emission tomography/computed tomography (^18^F-FDG PET/CT) in patients with locally advanced breast cancer (LABC). Methods: We retrospectively analyzed 158 LABC patients with FDG-avid, pathology-proven, metastatic ALN who underwent neoadjuvant chemotherapy (NAC) and curative surgery. Tumor and ALN texture parameters were extracted from pretreatment ^18^F-FDG PET/CT using Chang-Gung Image Texture Analysis software. The least absolute shrinkage and selection operator regression was performed to select the most significant predictive texture parameters. The predictive impact of texture parameters was evaluated for both progression-free survival and pathologic NAC response. Results: The median follow-up period of 36.8 months and progression of disease (PD) was observed in 36 patients. In the univariate analysis, ALN textures (minimum standardized uptake value (SUV) (*p* = 0.026), SUV skewness (*p* = 0.038), SUV bias-corrected Kurtosis (*p* = 0.034), total lesion glycolysis (*p* = 0.011)), tumor textures (low-intensity size zone emphasis (*p* = 0.045), minimum SUV (*p* = 0.047), and homogeneity (*p* = 0.041)) were significant texture predictors. On the Cox regression analysis, ALN SUV skewness was an independent texture predictor of PD (*p* = 0.016, hazard ratio 2.3, 95% confidence interval 1.16–4.58). Conclusions: ALN texture feature from pretreatment ^18^F-FDG PET/CT is useful for the prediction of LABC progression.

## 1. Introduction

Neoadjuvant chemotherapy (NAC) has been a widely used treatment option for locally advanced breast cancer (LABC). NAC has the advantages of downstaging with change to the operable status and response monitoring with change regimen for patients with unfavorable status [1]. However, it is difficult to predict treatment response and survival due to the heterogeneity of breast cancer. Thus, the accurate prediction of tumor response to NAC and survival is important for the selection of the optimal available therapy options.

^18^F-fluorodeoxyglucose positron emission tomography/computed tomography (FDG PET/CT) is a promising imaging modality used to characterize the metabolic status of tumors and provide prognostic information on the treatment response of cancer. In breast cancer, standardized uptake value (SUV), metabolic tumor volume (MTV), total lesion glycolysis (TLG), and their percent reduction changes have been associated with prognosis and evaluated treatment responses to NAC [2,3,4]. In addition to conventional analysis, texture analysis from pretreatment ^18^F-FDG PET/CT has recently shown emerging results in the predictive value of survival [5,6,7]. However, previous studies have focused on interval changes in conventional image features between baseline and interim/post-treatment ^18^F-FDG PET/CT or primary tumor texture parameters extracted from ^18^F-FDG PET/CT.

Although tumor size and axillary LN metastasis are clinically known to be very important prognostic factors in breast cancer, conventional PET radiomics analysis mainly evaluated the heterogeneity of primary tumor metabolism. Therefore, we intend to investigate whether there is a clinical impact by inserting the axillary lymph node as a radiomic s analysis parameter. To the best of our knowledge, the prognostic impact of PET-based axillary lymph node (ALN) texture features from pretreatment ^18^F-FDG PET/CT for the prediction of progression-free survival (PFS) has not been investigated in LABC with NAC.

In this study, we assessed the clinical value of the texture parameters of ALN extracted from pretreatment ^18^F-FDG PET/CT to predict PFS and pathologic response in LABC with NAC.

## 2. Materials and Methods

### 2.1. Patients

We retrospectively performed the analysis of medical data of LABC patients between January 2012 and December 2020 (age range, 26–70 years; mean age, 45.6 ± 9.2 years). All patients had conventional mammography, breast ultrasonography, CT or magnetic resonance imaging (MRI), and ^18^F-FDG PET/CT for staging workup. Neoadjuvant chemotherapy was performed with six cycles of doxorubicin plus cyclophosphamide (AC) or doxorubicin plus docetaxel (AT) regimens. Among pathological stage II and III breast cancer patients without distant metastasis who received mastectomy or breast-conserving surgery with ALN dissection, the patients confirmed as axillary lymph node metastasis were enrolled in this study. The inclusion criteria were as follows: (1) patients with LABC who have undergone pretreatment ^18^F-FDG PET/CT and NAC; (2) pathology-proven metastatic ALN with FDG-avid, short diameter > 1 cm, maximum standardized uptake value (SUVmax) > 1.0, and metabolic volume > 1.5 cm^3^; (3) no previous other malignant tumors including breast cancer history. Clinical data were collected including age at initial diagnosis, primary tumor size, 7th edition of the American Joint Committee on Cancer (AJCC) TNM stage, surgery method, and NAC regimen. Pathological data included estrogen receptor (ER), progesterone receptor (PR) expression, human epidermal growth factor receptor 2 (HER2) expression, and postoperative pathological TNM stage. Pathologic complete response (pCR) was defined as the absence of residual disease or the presence of residual ductal carcinoma in situ and without residual lymph node metastasis (ypT0/is and ypN0). In this study, patients with pCR were considered responders, and patients without pCR were considered non-responders. Progression-free survival (PFS) time was defined as the time from the date of diagnosis to the date of disease progression or last follow-up.

This study was conducted according to the guidelines of the Declaration of Helsinki and was approved by the Institutional Review Board of Soonchunhyang University Hospital Bucheon (IRB No. 2020-10-006). The requirement for informed patient consent was waived due to its retrospective design.

### 2.2. ^18^F-FDG PET/CT Acquisition

All patients fasted for more than 6 h before ^18^F-FDG PET/CT. The blood glucose level was controlled below 200 mg/dL. After intravenous injection of 370–550 MBq of ^18^F-FDG, torso PET and unenhanced CT images were acquired using two dedicated PET/CT scanners (Discovery STe, GE Healthcare, Waukesha, WI, USA, and Biograph 128mCT, Siemens Medical Solutions, Knoxville, TN, USA). The 16-slice helical CT scan with 140 keV, 30–170 mAs, slice section of 3.75 mm and 100 keV and 50 mAs, slice section of 5 mm were acquired from vertex to mid-thigh. PET images were acquired in the same range of body and attenuation-corrected PET images (voxel size, 3.9 × 3.9 × 3.3 mm^3^) were reconstructed using a 3D-ordered subset-expectation maximization algorithm (20 subsets, 2 iterations).

### 2.3. Image Feature Extraction and Selection

For quantitative analysis, the volumes of interest (VOIs) from the primary tumor and ALN were semi-automatically delineated using the gradient-based algorithm (PET Edge) in MIM version 6.4 (MIM Software Inc., Cleveland, OH, USA). These VOIs were saved as a DICOM-RT structures that were imported into the Chang-Gung Image Texture Analysis toolbox (http://code.google.com/p/cgita, accessed on 1 March 2020) implemented in MATLAB software (version 2014b; MathWorks, Inc., Natick, MA, USA) to extract and analyze the texture indices from PET images. Based on the IBSI (Imaging Biomarker Standardization Initiative) guideline, forty-seven tumoral heterogeneity indices (5 Cooccurrence matrix, 5 Voxel-alignment matrix, 5 Neighborhood intensity difference matrix, 9 Intensity size-zone matrix, 1 Normalized cooccurrence matrix, 11 SUV statistics, 7 Texture feature coding cooccurrence matrix, 4 Neighborhood gray-level dependence) were extracted from CGITA software. To increase the reliability of the analysis, intensity-based statistics (11 SUV statistics) were extracted in ALN heterogeneity indices. The extracted tumor and ALN texture features were described in the Supplementary Data (Table S1). Subsequently, we used the least absolute shrinkage and selection operator (LASSO) regression algorithm with 10-fold cross-validation to select the most useful prognostic features based on the association between texture parameters and PFS of the patients among the extracted PET based texture features. Because LASSO can shrink the effect of unimportant features and can set their effects to zero together with removing redundancy among the features, we could obtain texture features with non-zero coefficients.

### 2.4. Statistical Analysis

Categorical variables of clinicopathologic and texture parameters on pathologic response were evaluated by a chi-squared test. Mann–Whitney test or independent t-test were used to analyze the statistical difference of continuous variables. Multivariate logistic regression analysis was performed to assess independent predictors of pathologic response among the variables that were significant in univariate logistic regression analysis. Receiver operating characteristic (ROC) curve analysis was performed and the optimal cutoff value of each parameter was determined by the area under the curve with the Youden index. Kaplan–Meier analysis and log-rank test were performed to compare survival between clinicopathologic and texture parameters. The effect of clinicopathologic and texture parameters on survival was analyzed by Cox proportional hazard method; *p*-values of <0.05 were defined as significant. Rex ver. 3.0.3 (RexSoft, Seoul, Korea) and MedCalc software package (Ver. 9.5, MedCalc Software, Mariakerke, Belgium) were used for the clinical and radiomic data analyses.

## 3. Results

### 3.1. Patient Characteristics and Pathologic Outcomes

**Figure 1:**
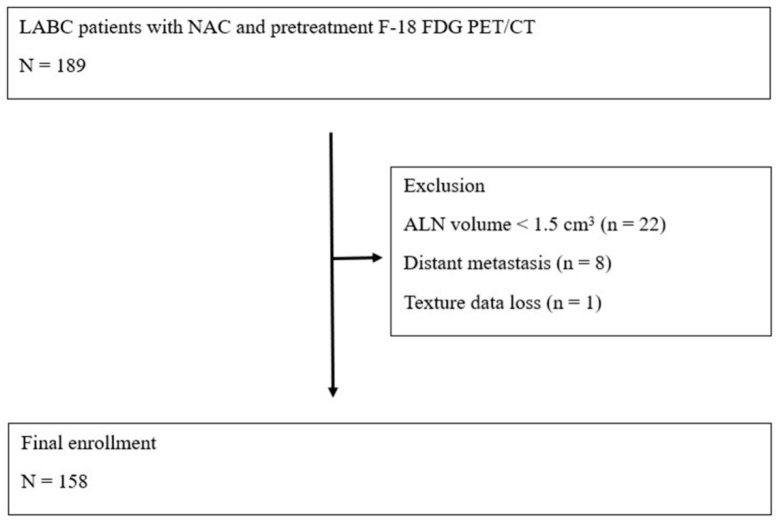
Patient enrollment flow chart.

The characteristics and enrollment of 158 patients are summarized in Table 1 and Figure 1. The median patient age was 46 (26–70) years, and the median primary tumor size was 4.0 cm (range 1.2–14.1 cm). Most patients (94.3%) had invasive ductal carcinoma. Clinical stage II was observed in 29 (18.4%) patients and stage III in 129 (81.6%) patients. Clinical subgroups were hormone receptor (HR)-positive/HER2-negative in 60 (38.0%) patients, HER2-positive in 49 (31.0%), and triple-negative in 49 (31.0%). Seventeen patients (10.8%) were treated with doxorubicin plus cyclophosphamide (AC) or doxorubicin plus docetaxel (AT) regimens, whereas 141 (89.2%) patients received doxorubicin plus cyclophosphamide (AT). Ninety-seven patients (61.4%) underwent breast-conserving surgery, whereas 61 patients (38.6%) underwent a mastectomy. According to the pathologic response status, 26 (16.4%) patients were responders, whereas 132 (83.6%) were non-responders. Disease progression was observed in 36 (22.8%) patients. A total of 14 patients (8.8%) received adjuvant chemotherapy with doxorubicin plus docetaxel after surgery.

### 3.2. PFS Analysis

**Figure 2:**
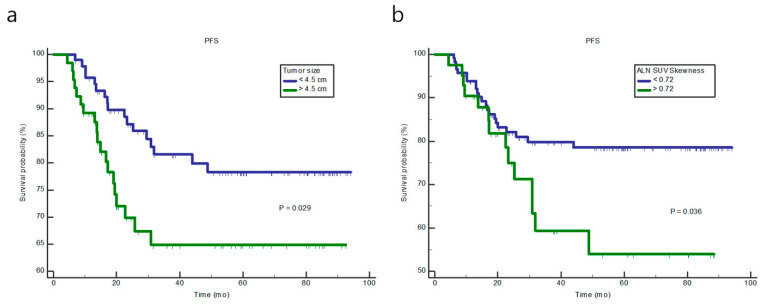
Kaplan–Meier analysis of progression-free survival according to tumor size (**a**) and axillary lymph node (ALN) skewness (**b**).

The median follow-up period was 36.8 (range, 6.1–94.1) months, and disease progression was observed in 36 patients. LASSO regression analysis revealed that three tumoral texture parameters (low-intensity size zone emphasis (LISZE), minimum SUV, and homogeneity) and four ALN texture parameters (SUV skewness, SUV bias-corrected kurtosis, minimum SUV, and TLG) were the most useful for predicting PFS. The Kaplan–Meier survival curves showed that patients with higher SUV skewness of ALN texture parameter and tumor size had a significantly poorer PFS (*p* = 0.036 and 0.029, respectively, Figure 2). When Cox proportional hazard regression analysis was performed, ALN SUV skewness was the only independent texture predictor of disease progression (*p* = 0.016, HR 2.3, 95% CI 1.16–4.58, Table 2).

To evaluate the additional prognostic performance of the ALN texture parameter, we developed combined models according to the selected significant features from the LASSO regression in a model I (clinical feature (tumor size) + tumor texture features (LISZE, minimum SUV, homogeneity)) and model II (clinical feature (tumor size) + tumor texture features (LISZE, minimum SUV, homogeneity) + ALN texture features (SUV skewness, SUV bias-corrected kurtosis, minimum SUV, and TLG)). Combination model II showed significantly higher concordance index (C-index) than combination model I (C-index 0.66 and 0.58, *p* = 0.002, respectively).

**Figure 3:**
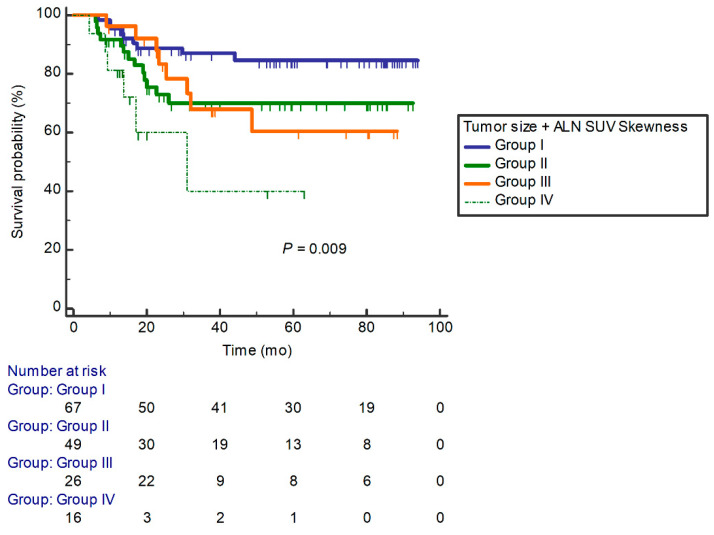
Combination model of clinical and ALN texture parameters for progression-free survival.

In addition, we divided the four groups according to the combinations of ALN SUV skewness and tumor size to evaluate additional detailed survival efficacy in each group (group I, low ALN SUV skewness and low tumor size; group II, low ALN SUV skewness and high tumor size; group III, high ALN SUV skewness and low tumor size; and group IV, high ALN SUV skewness and high tumor size). The median PFS duration of group IV was 33.9 months. Patients with a high ALN SUV skewness and high tumor size (Group IV) showed significantly shorter PFS than the other groups (*p* = 0.009, Figure 3).

### 3.3. Correlation between Texture Parameters and Pathologic Response

Among the texture parameters of pretreatment PET, six tumoral texture parameters (correlation (*p* = 0.049), minimum SUV (*p* = 0.015), LISZE (*p* = 0.040), Size Zone Variability (*p* = 0.038), intensity variability (*p* = 0.042), strength (*p* = 0.039)) and 1 ALN texture parameter (SUV kurtosis (*p* = 0.037)) showed a significant difference between responders and non-responders. In the analysis of clinicopathologic features such as cT and ypT status, ER, PR, and HER2 neu hormonal receptor status and surgical method were significant differences between the two groups. Smaller tumor size, breast-conserving surgery, and negative ER and PR status occupied more distribution in the responder group. Data distribution and characteristics of the two groups are summarized in Table 3.

In multivariate logistic regression analysis, the minimum SUV of the tumor texture parameter was the only independent predictor of pathologic response (*p* = 0.024, OR 6.82, 95% CI 1.97–23.65, Table 4 and Table 5).

## 4. Discussion

A limitation of existing PET radiomics studies in breast cancer is that they were limited to primary tumors, although both tumor and axillary LN metastasis are clinically important.

Therefore, the unique point of our study is to analyze both tumor and axillary LN metastasis. Because axillary LN is small in size, tumor intensity and heterogeneity analyzed in general PET radiomics can reduce the reliability of the analysis results [8]. To increase the reliability of the analysis, intensity-based statistics (11 SUV statistics) were extracted in axillary LN texture features, unlike tumors.

When analyzing by adding various SUV statistics features in addition to SUVmax, TLG, and tumor volume used in existing PET-based clinical studies, the expected clinical impact of this analysis lies in discovering new SUV statistics parameters that can better reflect prognosis.

This study suggests the ALN texture parameter (SUV skewness) measured in pretreatment ^18^F-FDG PET/CT was an independent predictor for the PFS in LABC with NAC. The relationship between conventional or volumetric PET parameters and other prognostic factors has been reported in breast cancer [9,10]. To overcome the limitation of conventional PET parameters that cannot reflect tumor heterogeneity, texture analysis-extracted baseline or post-treatment ^18^F-FDG PET/CT were reported as predictors of survival [11,12,13]. ALN metastasis strongly affects the prognosis of recurrence; thus, its metabolic or texture characteristics are important when making clinical decisions for treatment regimens and prognostic assessment in breast cancer [14,15]. However, the prognostic impact of pretreatment PET-based texture parameters of ALN for predicting disease progression in LABC has not been directly reported yet. SUV skewness shows the asymmetry of the distribution of lesion intensity about its mean value, and a higher SUV skewness reflects the statistical heterogeneity of images [16]. In previous studies, tumor SUV skewness was a useful predictor for LN metastasis in the uterine cervix, lung, pancreas, brain, and nasopharyngeal cancers [17,18,19,20]. Another study reported that tumor SUV skewness was associated with PFS and OS in lymphoma [21,22]. In a previous study on breast cancer, an increase in tumor SUV skewness was associated with poor OS and PFS [23]. However, in another study on breast cancer, tumor SUV skewness was not an independent predictor of PFS [24]. Compared with previous studies, the present study focused on the prognostic value of the ALN texture parameter, not the status of ALN metastasis; thus, we suggested that ALN SUV skewness was a significant predictor of disease progression. Patients with high SUV skewness of ALN showed a worse prognosis than patients with low SUV skewness of ALN. This may indicate the importance of PET-derived metastatic ALN heterogeneity as a predictor of disease progression in LABC. Compared to the model combining clinicopathologic and tumoral texture parameters, it is expected that adding the ALN texture parameter will further increase predictive performance, providing additional prognostic information. Furthermore, considering that tumor size is a well-known prognostic factor of breast cancer, in the combination of clinical and texture parameters, the high SUV skewness of ALN and high tumor size group showed shorter survival days and poor prognosis for PFS. Regardless of the tumor size, the higher ALN SUV skewness group showed a worse prognosis. The combination of ALN texture and clinical parameters is expected to stratify the detailed prognosis in LABC with NAC.

Of the PET-derived texture parameters, six tumoral parameters and one ALN texture parameter showed significant differences between responders and non-responders. However, the ALN texture parameter did not remain in the multivariate logistic regression analysis. On the other hand, the minimum SUV of the primary tumor parameter remained an independent predictor of pathologic response in multivariate analysis. Tumors with low tumoral minimum SUV showed less pathologic complete response than tumors with high tumoral minimum SUV. SUV kurtosis reflects the peaked or flat distribution of image gradient and increases with higher heterogeneity. [25]. In a previous study, tumoral SUV kurtosis showed a significant predictive value for post-treatment response in rectal cancer [26]. In another study, tumoral SUV kurtosis showed a significant difference between primary colon cancer and hepatic metastasis [27]. In the present study, we focused on the predictive impact of ALN texture itself, where previous studies reported that tumoral SUV kurtosis was associated with response prediction. Although the multivariate results were not significant, ALN SUV kurtosis had a discriminative tendency of treatment response and showed lower ALN SUV kurtosis in responders.

In this study, the inclusion criterion of ALN status was pathology-proven metastasis with FDG-avid, short diameter >1 cm, SUVmax >1.0, and metabolic volume >1.5 cm^3^. Previous studies have reported that short diameter of 5 mm and SUVmax 2.0 were widely used as judgment indicators of ALN status because of their best sensitivity and specificity of the results, highest diagnostic accuracy, and reproducibility [28,29]. Of all the patients, nine had ALN SUVmax <2.0 (range 1.58–1.99) and half of them ≥1.9; thus, the effect on the analysis results is considered negligible in this study. Other studies have reported that lesions with volumes and dimensions of at least equal to three times the spatial resolution of the scanner (about 5 mm) were included to reduce the effect of partial volume effect on texture calculations [30]. In this study, only the lesions with a volume of >1.5 cm^3^ were included. Based on previous studies, our criteria for ALN status are not too unreasonable.

This study has some limitations. First, this study was conducted retrospectively, and the results might be affected by selection bias. Second, given the limited resolution of PET, the metabolic texture parameters of either small lesion such as ALN could be underestimated. Third, the results from acquired images using two different PET/CT scanners are likely to be inconsistent. Nevertheless, acquired texture parameters are considered to be consistent because of the use of mathematical relationships among voxels and PET Edge technique with different reconstruction algorithms, imaging techniques, sphere diameter effects, and good reliability of the texture analysis [12,31,32]. Finally, although our data were analyzed using 10-fold cross-validation and LASSO regression to overcome overfitting, external validation is required for supporting the clinical significance. In the future, further controlled prospective large-scale studies are needed to verify the more accurate and generalized predictive value of the texture-based analysis of ^18^F-FDG PET/CT.

## 5. Conclusions

PET-based texture analysis of ALN on pretreatment ^18^F-FDG PET/CT could predict disease progression in LABC with NAC. High ALN SUV skewness was an independent prognostic factor associated with poor PFS.

## Figures and Tables

**Table 1:** Clinicopathologic characteristics.

Variables	Total (n = 158)
Age (median, years, range)	46 (26–70)
Tumor size (median, cm, range)	4 (1.2–14.1)
cT status	
cT1–2	94 (59.4%)
cT3–4	64 (40.6%)
cN status	
cN1	42 (26.5%)
cN2–3	116 (73.5%)
ypT status	
ypT0	20 (12.6%)
ypTis	15 (9.5%)
ypT1–2	94 (59.5%)
ypT3–4	22 (19.4%)
ypN status	
ypN0	57 (36.1%)
ypN1	56 (35.4%)
ypN2–3	45 (28.5%)
Clinical AJCC stage	
II	29 (18.4%)
III	129 (81.6%)
Histology	
IDC ^a^	149 (94.3%)
Non-IDC	9 (5.7%)
ER ^b^	
Negative	77 (48.7%)
Positive	81 (51.3%)
PR ^c^	
Negative	98 (62.0%)
Positive	60 (38.0%)
HER2 ^d^ neu	
Negative	110 (69.6%)
Positive	48 (30.4%)
Breast surgery	
Mastectomy	61 (38.6%)
BCS ^e^	97 (61.4%)
NAC ^f^ regimen	
Anthracycline-based	17 (10.7%)
Taxene-based	141 (89.3%)
Treatment response	
pCR ^g^	26 (16.4%)
non-pCR	132 (83.6%)
Progression of disease (PD)	
Non-PD	122 (77.2%)
PD	36 (22.8%)
Adjuvant chemotherapy	
Taxene-based	14 (8.8%)

^a^ IDC = invasive ductal carcinoma, ^b^ ER = estrogen receptor, ^c^ PR = progesterone receptor, ^d^ HER2 = human epidermal growth factor receptor-2, ^e^ BCS = breast-conserving surgery, ^f^ NAC = neoadjuvant chemotherapy, ^g^ pCR = pathologic complete response.

**Table 2:** Univariate and multivariate analyses of predictive factors for progression-free survival.

Parameter	Univariate		Multivariate	
	HR ^a^ (95% CI ^b^)	*p* Value	HR (95% CI)	*p* Value
Tumor size	2.04 (1.05–3.93)	0.029	2.30 (1.18–4.49)	0.014
Clinical Stage	2.06 (0.91–4.68)	0.082		
ER ^c^	0.76 (0.39–1.46)	0.412		
PR ^d^	0.52 (0.26–1.01)	0.056		
HER2 neu ^e^	0.74 (0.36–1.50)	0.413		
LISZE ^f^ tumor	0.77 (0.40–1.48)	0.434		
Minimum SUV ^g^ tumor	1.61 (0.81–3.18)	0.166		
Homogeneity tumor	0.81 (0.41–1.60)	0.557		
SUV BC ^h^ kurtosis ALN ^i^	2.11 (0.93–4.89)	0.064		
SUV skewness ALN	2.01 (1.03–3.95)	0.036	2.31 (1.16–4.58)	0.016
Minimum SUV ALN	1.69 (0.74–3.86)	0.206		
TLG ^j^ ALN	1.95 (0.89–4.30)	0.087		

^a^ HR = hazard ratio, ^b^ CI = confidence interval, ^c^ ER = estrogen receptor, ^d^ PR = progesterone receptor, ^e^ HER2 = human epidermal growth factor receptor-2, ^f^ LISZE = low-intensity size zone emphasis, ^g^ SUV = standardized uptake value, ^h^ BC = bias-corrected, ^i^ ALN = axillary lymph node, ^j^ TLG = total lesion glycolysis.

**Table 3:** Characteristics of responders and non-responders.

	Responders (n = 26)	Non-Responders (n = 132)	*p* Value
Age (median, years, range)	49 (32–58)	45 (25–70)	0.156
Tumor size (median, cm, range)	2.9 (1.4–11.0)	4.5 (1.2–14.1)	0.003
cT status			0.002
cT1–2	22 (84.6%)	69 (52.2%)	
cT3–4	4 (15.4%)	63 (47.8%)	
cN status			0.271
cN1	9 (34.6%)	32 (24.2%)	
cN2–3	17 (63.4%)	100 (75.8%)	
ypT status			0.001
ypT0	15 (57.7%)	5 (19.2%)	
ypTis	11 (42.3%)	4 (3.0%)	
ypT1–2	0	94 (71.2%)	
ypT3–4	0	29 (6.6%)	
Clinical AJCC stage			0.075
II	8 (30.7%)	21 (15.9%)	
III	18 (69.3%)	111 (84.1%)	
Histology			0.199
IDC ^a^	26 (100%)	124 (93.9%)	
Non-IDC	0	8 (6.1%)	
ER ^b^			0.038
Negative	17 (65.4%)	57 (43.2%)	
Positive	9 (34.6%)	75 (56.8%)	
PR ^c^			0.004
Negative	22 (84.6%)	72 (54.5%)	
Positive	4 (15.4%)	60 (45.5%)	
HER2 ^d^ neu			0.003
Negative	11 (42.3%)	95 (71.9%)	
Positive	15 (57.7%)	37 (28.1%)	
Breast surgery			0.026
Mastectomy	5 (19.2%)	56 (42.4%)	
BCS ^e^	21 (80.8%)	76 (57.6%)	
NAC ^f^ regimen			0.888
Anthracycline-based	3 (11.5%)	14 (10.6%)	
Taxene-based	23 (88.5%)	118 (89.4%)	
Progression of disease (PD)			0.007
Non-PD	25 (96.1%)	94 (71.2%)	
PD	1 (3.9%)	38 (28.8%)	
Tumor texture index			
Correlation	0.68 (0.43–0.83)	0.73 (0.32–0.89)	0.049
Minimum SUV ^g^	1.23 (0.05–1.92)	0.72 (0.01–2.88)	0.015
LISZE ^h^	0.02 (0.00–0.06)	0.01 (0.00–0.06)	0.040
SZV ^i^	73.39 (24.60–678.15)	131.73 (18.10–1784.40)	0.038
Intensity variability	4.92 (1.66–4131.62)	12.62 (1.21–66237.00)	0.042
Strength	53.61 (5.42–127.65)	35.37 (2.93–132.34)	0.039
ALN ^j^ texture index			
SUV Kurtosis	2.17 (1.6–9.6)	2.62 (1.4–9.0)	0.037

^a^ IDC = invasive ductal carcinoma, ^b^ ER = estrogen receptor, ^c^ PR = progesterone receptor, ^d^ HER2 = human epidermal growth factor receptor-2, ^e^ BCS = breast-conserving surgery, ^f^ NAC = neoadjuvant chemotherapy, ^g^ SUV = standardized uptake value, ^h^ LISZE = low-intensity size zone emphasis, ^i^ SZV = size zone variability, ^j^ ALN = axillary lymph node.

**Table 4:** ROC analysis of the significant clinical and textural parameters to identify pathologic complete response.

Parameters	Cutoff	AUC ^a^	Sn ^b^ (%)	Sp ^c^ (%)	*p* Value
SUV ^d^ Kurtosis ALN ^e^	<2.387	0.630	69.2	61.7	0.034
Tumor size	<2.900	0.682	61.5	74.0	0.002
Correlation tumor	<0.718	0.622	73.1	57.8	0.041
IV ^f^ tumor	<6.037	0.626	76.9	47.7	0.040
LISZE ^g^ tumor	>0.009	0,628	76.9	49.2	0.029
Minimum SUV tumor	>1.180	0.651	57.7	82.8	0.021
SZV ^h^ tumor	<76.95	0.629	57.7	67.2	0.028
Strength tumor	>48.51	0.628	57.7	69.5	0.034

^a^ AUC = area under curve, ^b^ Sn = sensitivity, ^c^ Sp = specificity, ^d^ SUV = standardized uptake value, ^e^ ALN = axillary lymph node, ^f^ IV = intensity variability, ^g^ LISZE = low-intensity size zone emphasis, ^h^ SZV = size zone variability.

**Table 5:** Multivariate regression analysis of the clinical and textural parameters for the prediction of pathologic complete response.

Parameters	OR ^a^	95% CI for OR		*p* Value
		Lower	Upper	
SUV ^b^ kurtosis ALN ^c^	1.317	0.503	3.449	0.573
Tumor size	0,216	0.067	0.695	0.010
Correlation tumor	0.914	0.155	5.359	0.920
IV ^d^ tumor	1.651	0.306	8.909	0.559
LISZE ^e^ tumor	0.695	0.180	2.676	0.596
Minimum SUV tumor	6.829	1.971	23.659	0.024
SZV ^f^ tumor	1.680	0.180	15.605	0.648
Strength tumor	1.514	0.150	15.241	0.724

^a^ OR = odds ratio, ^b^ SUV = standardized uptake value, ^c^ ALN = axillary lymph node, ^d^ IV = intensity variability, ^e^ LISZE = low-intensity size zone emphasis, ^f^ SZV = size zone variability.

## Data Availability

The data that support the findings of this study are available on request from the corresponding author. The data are not publicly available due to privacy or ethical restrictions.

## Supplementary Materials

The following supporting information can be downloaded at: https://www.mdpi.com/article/10.3390/diagnostics12102285/s1, Table S1: PET-based texture features extracted from CGITA.
