# Supplementary material for: Prognostic Value of Axillary Lymph Node Texture Parameters Measured by Pretreatment 18F-Fluorodeoxyglucose Positron Emission Tomography/Computed Tomography in Locally Advanced Breast Cancer with Neoadjuvant Chemotherapy
Source: Diagnostics (Basel). 2022 Sep 22;12(10):2285. doi: 10.3390/diagnostics12102285 (PMC9600297; doi:10.3390/diagnostics12102285)
Supplement: Supplementary file 1 [file diagnostics-12-02285-s001.zip › diagnostics-1866826-supplementary.pdf]

Table S1. PET-based texture features extracted from CGITA.

Feature family of IBSI [a]	Parent matrix of CGITA [b]	Texture features of CGITA
Tumor texture features		
Cooccurrence matrix	Cooccurrence	Second angular moment
		Contrast
		Entropy
		Dissimilarity
		Inverse difference moment
Run length matrix	Voxel-alignment	Long run emphasis
		Intensity variability
		Run length variability
		Run percentage
		High intensity run emphasis
Neighborhood gray tone difference	Neighborhood intensity difference	Coarseness
		Contrast
		Busyness
		Complexity
		Strength
Intensity size zone matrix	Intensity size zone	Short zone emphasis
		Large zone emphasis
		Intensity variability
		Size zone variability
		High intensity zone emphasis
		Low intensity short zone emphasis
		High intensity short zone emphasis
		Low intensity large zone emphasis
		High intensity large zone emphasis
Cooccurrence matrix	Normalized cooccurrence	Correlation
Intensity based statistics	SUV statistics	Minimum SUV
		Maximum SUV
		Mean SUV
		SUV Variance
		SUV SD [c]
		SUV Skewness
		SUV Kurtosis
		SUV bias corrected Skewness
		SUV bias corrected Kurtosis
		TLG [d]
		Tumor volume
Morphologic feature matrix	Texture feature coding cooccurrence	Second angular moment
		Contrast
		Entropy
		Homogeneity
		Intensity
		Inverse difference moment
		Code entropy
Neighboring gray level dependence	Neighboring gray level dependence	Small number emphasis
		Large number emphasis
		Number nonuniformity
		Entropy
ALN texture features		
Intensity based statistics	SUV statistics	Minimum SUV
		Maximum SUV
		Mean SUV
		SUV Variance
		SUV_SD

		SUV Skewness
		SUV Kurtosis
		SUV bias corrected Skewness
		SUV bias corrected Kurtosis
		TLG
		Tumor volume

[a] IBSI = Imaging Biomarker Standardization Initiative, [b] CGITA = Chang-Gung Image Texture Analysis toolbox, [c] SD = standard deviation, [d] TLG = total lesion glycolysis.
